# A Review: Point Cloud-Based 3D Human Joints Estimation

**DOI:** 10.3390/s21051684

**Published:** 2021-03-01

**Authors:** Tianxu Xu, Dong An, Yuetong Jia, Yang Yue

**Affiliations:** 1 Institute of Modern Optics, Nankai University, Tianjin 300350, China; 1120180105@mail.nankai.edu.cn (T.X.); 1120190109@mail.nankai.edu.cn (D.A.); 1911343@mail.nankai.edu.cn (Y.J.); 2 Angle AI (Tianjin) Technology Company Ltd., Tianjin 300450, China

**Keywords:** point cloud, joint estimation, skeleton extraction, depth sensor, skeleton tracking, computer vision, human representation, convolutional neural network, random tree walk, random forest, geodesic features, global features, deformation model, hand pose tracking, action recognition

## Abstract

Joint estimation of the human body is suitable for many fields such as human–computer interaction, autonomous driving, video analysis and virtual reality. Although many depth-based researches have been classified and generalized in previous review or survey papers, the point cloud-based pose estimation of human body is still difficult due to the disorder and rotation invariance of the point cloud. In this review, we summarize the recent development on the point cloud-based pose estimation of the human body. The existing works are divided into three categories based on their working principles, including template-based method, feature-based method and machine learning-based method. Especially, the significant works are highlighted with a detailed introduction to analyze their characteristics and limitations. The widely used datasets in the field are summarized, and quantitative comparisons are provided for the representative methods. Moreover, this review helps further understand the pertinent applications in many frontier research directions. Finally, we conclude the challenges involved and problems to be solved in future researches.

## 1. Introduction

**Figure 1:**
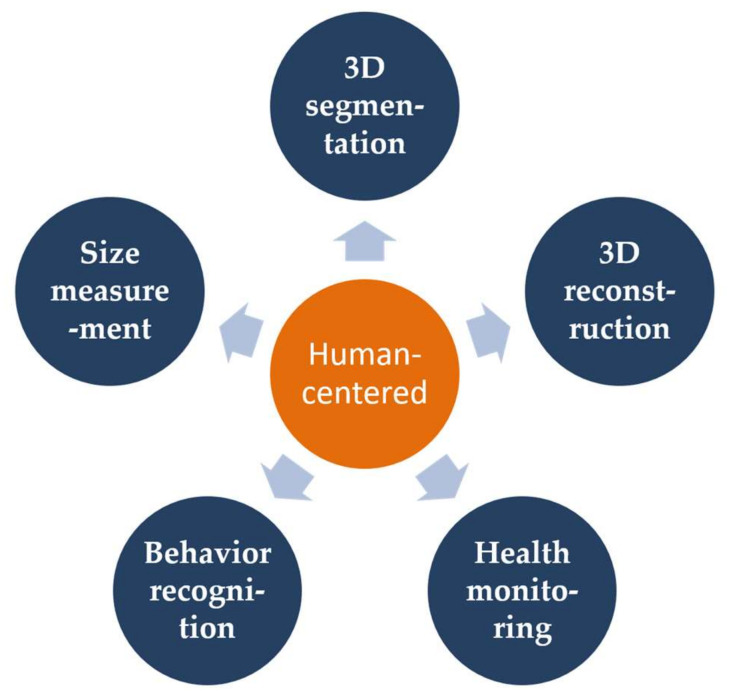
Human-centered applications of depth sensor.

The depth camera can provide the ranging information from a single depth image or a point cloud for a variety of applications, such as gaming, three-dimensional (3D) reconstruction and object recognition. Many human-centered tasks based on depth camera had been investigated in the last few years, as shown in Figure 1. For example, 3D human reconstruction is the process of recovering a 3D human surface model by finding the accurate correspondence between frames [1,2]. The 3D segmentation technology of human body is the most critical technology in applications such as digital clothing and computer animation [3]. The health monitoring system using the depth information can check the diseased parts of the human body to facilitate the guidance of rehabilitation training [4]. The size measurement of human body based on the depth camera is a safe and non-contact fast measurement method, which overcome the challenges of high cost and bulky electronic scanners [5]. Human behavior recognition, as a fundamental research problem, is an extremely significant component and extensively studied research subject in computer vision [6]. The ultimate objective of encoding human body is to extract the various joints of a predefined skeleton in a simplified manner. 

Conventional methods for detecting joints of human body utilize two-dimensional (2D) images or video, taken by traditional cameras. Significant progress has been made for these methods in recent years, by leveraging the powerful deep learning. However, there are still some limitations on human pose estimation using only 2D images, due to the coexisting complex backgrounds, variable viewpoints, highly flexible poses, etc. Additional depth information can provide enriched 3D data to overcome the limitation of 2D data. 

**Figure 2:**
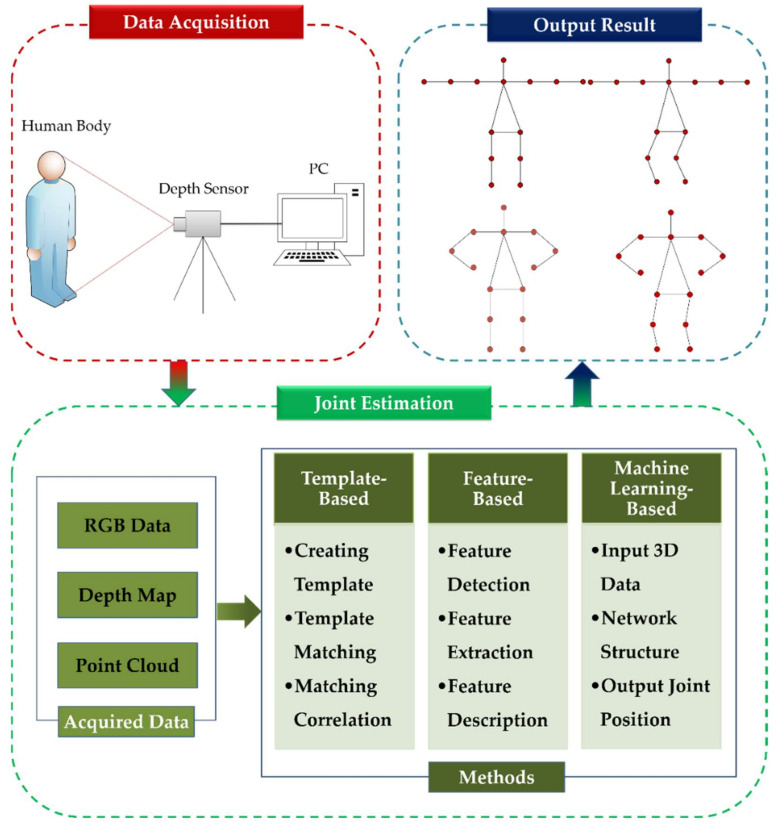
The flow diagram of point cloud-based 3D joints extraction.

The purpose of depth camera-based 3D human pose estimation is to locate the (*x*, *y*, *z*) coordinates of joints in 3D space. Ideally, once the captured human pose changes, the joints can still be reliably estimated. Figure 2 describes the specific overview of the 3D joints extraction. The first step of this process is to capture the human poses by a depth sensor. Since the obtained poses of human body contain redundant information, it is necessary to process the data in advance. Next, the pre-processed data is used to calculate the 3D coordinates of the joints using special methods, such as template-based method, feature-based method, and machine learning-based method. Finally, the error analysis is performed. 

Usually 3D data formats include depth map, point cloud, mesh, and voxel grid, etc. Here a point cloud is a collection of points in the 3D space. In addition to the commonly used 3D coordinate information, point cloud can also carry other dimensional information such as color and normal vector. The point cloud can be obtained directly through the depth sensors. Compared with the voxel grid, the storage space of the point cloud is smaller, and the geometric information can still be expressed well after the rotation. Compared with the mesh method, the point cloud is easily obtained, while there is not direct method to acquire the mesh data. Compared with the depth map, the point cloud represents the 3D object in a more intuitive way. Moreover, the conversion between the point cloud and the other 3D formats is quite straight forward. The widely used open source libraries for 3D point cloud processing are mainly Point Cloud Library (PCL) [7] and Open3D [8]. PCL is a cross-platform C++ library, which implements a large number of point cloud-related general algorithms and efficient data structures, involving point cloud acquisition, filtering, segmentation, registration, retrieval, feature extraction, recognition, tracking, surface reconstruction, visualization, etc. It can support multiple operating systems such as Windows, Linux, Android, Mac OS X, and some embedded real-time systems. Open3D is a modern library that can support the rapid software development for 3D data processing. A set of data structures and algorithms are exposed in C++ and Python, its core features include 3D data structure, 3D data processing algorithms, scene reconstruction, and 3D visualization. PCL is more mature with a large number of data structures and algorithms for 3D data processing. In contrast, Open3D can be installed and used in the python environment, and the programming is faster and simpler. 

**Figure 3:**
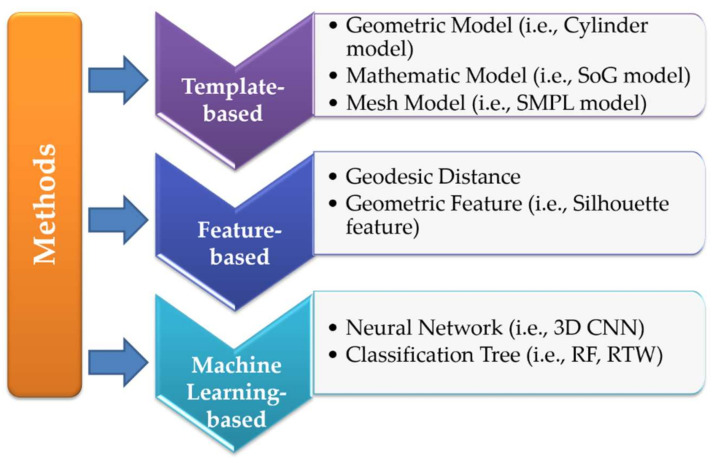
Categorization of the methods for the 3D joints extraction based on point cloud. SoG is sums of spatial gaussians; SMPL is skinned multi-person linear; CNN is convolutional neural network; RF is random forest; RTW is random tree walk.

The aim of this survey is to provide a comprehensive overview of 3D human joints extraction based on point cloud. The survey mainly focuses on publication of point cloud-based joint estimation for human body in computer vision. Here, the point cloud of human body is directly captured from 3D ranging devices. The involved content mainly includes the datasets of relevant pose, the methods of point cloud-based joint extraction, and the applications of point cloud-based joint extraction. The summarized methods are shown in Figure 3, including template-based methods, feature-based methods and machine learning-based methods. Compared with the existing surveys, the main contributions of this review include:

1. To the best of our knowledge, this is the first review to summarize the point cloud-based 3D joints estimation of human body, thereby providing readers with a complete overview of the latest researches and developments in the field.
2. The review categorizes the advanced methods based on their working principles in a comprehensive way, and we enumerate some milestone works in recent years.
3. The datasets and applications of point cloud-based 3D joint extraction are analyzed. In addition, the results from different literatures are summarized and compared.

The remainder of this review is organized as following: Section 2 introduces sets of devices for range detection; Section 3 reviews the existing point cloud-based methods of human joints extraction; Section 4 enumerates the 3D human dataset; Section 5 discusses various applications of point cloud-based joint extraction; Section 6 concludes this survey with potential research directions in the future.

## 2. Depth Sensors for 3D Data Acquisition

**Figure 4:**
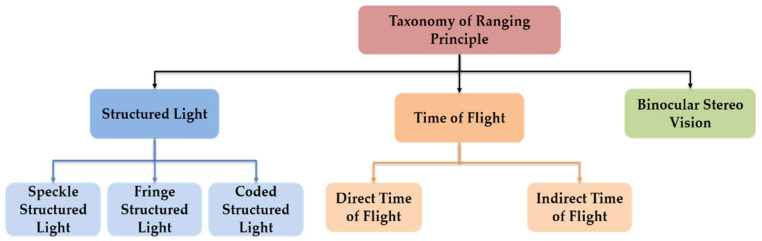
Classification of depth sensors for 3D data acquisition.

According to the ranging principle, we divide the depth sensors into three categories as shown in Figure 4, binocular stereo vision, time-of-flight (ToF) and structured light technologies. Early research focused on the passive method, such as binocular stereo vision, to calculate the depth information. Typically, two cameras were used to take pictures of the object from different perspectives. This mechanism is similar to imaging by two eyes of human. However, the result can be easily affected by the texture of object, and it is also time-consuming in the registration process. Compared with the passive ranging method, rapid active ranging shows obvious advantages. On one hand, radar and Lidar are commonly used in military to reconnoiter and detect battlefields in various environments, and it is also manipulated for obstacle detection in automatic driving technology. On the other hand, with the commercial application of low-cost depth cameras, the related research based on depth cameras has gradually unlocked new applications in the mobile and intelligent terminal devices.

The ToF depth camera first continuously sends light pulses to the detected object, and then the sensor is used to receive the light returned from the object. The final distance is calculated when the flight (round trip) time of the detected light pulse is obtained. ToF sensors are divided into two types: direct time-of-flight (dToF) and indirect time-of-flight (iToF) sensors according to different modulation methods. Lighting units of dToF generally use LEDs or lasers, including laser diodes and vertical cavity surface emitting lasers (VCSELs), to emit high-performance pulsed light, which directly measures the time difference from the emitter to the receiver and multiplies it by the speed of light to measure the relative distance of the object. The receiver must be a special sensor with very high accuracy, so it is difficult to reduce the cost and miniaturization. The light emitted by the iToF sensor is modulated by a continuous wave, whose intensity changes regularly. According to the selection of detected distance, it can be divided into pulsed light and continuous light. Next, the iToF depth cameras compare the signal difference between the emitted signal and the reflected signal, and then multiply it by the speed of light to get the relative distance. In addition, the response speed of the iToF receiver is not as fast as dToF, and it cannot accurately sense for the sub-nanosecond time difference. Fundamentally, radar and Lidar are also a kind of ToF sensor. The former uses millimeter waves, and has stronger anti-interference ability, the latter emits laser signals, and has higher detection accuracy. Structured light cameras project the invisible pseudo-random light spots to the detected object through the infrared (IR) emitter. According to the produced different light spots, they can be divided into speckle structured light, fringe structured light, and coded structured light cameras. The projected light spot is unique and known in the spatial distribution, and have been pre-stored in the structured light memory. The size and shape of these speckles projected on the observed object vary according to the distance and direction of the object and the camera. The captured spots are compared with the known spots, and then the depth information is obtained. Different depth cameras can be selected according to specific parameters, and the detailed comparison is shown in Table 1.

## 3. Methods of Point Cloud-Based Joint Estimation

This section mainly describes several methods to achieve human joint extraction. Existing surveys have made qualitative comparisons of the joint extraction techniques from the perspective of RGB or depth map. We limit our attention to point cloud-based methods, which are mainly divided into feature-based methods, template-based methods and machine learning-based methods. Each method is discussed in each section, respectively. 

### 3.1. Template-Based Methods

The human body is a flexible and complex object with many specific features, such as movement structure, body shape, surface texture, body parts or joint positions. A mature human model is not necessary to contain all the human attributes. Otherwise, it should meet the specific task of combining and describing human poses. The template-based method is intuitive and simple. It judges the motion category by comparing the similarity between the detected object and the constructed template. The existing template-based algorithms can be roughly divided into three categories according to their principles, including geometric model, mathematical model, and mesh model. When the model is selected to match with the observed point cloud of the human body, the joints are regarded as the connection of the rigid part to achieve pose estimation. A complex model usually features more characteristic parameters; it can provide a better approximation for the human body to achieve improved reality and accuracy.

#### 3.1.1. Geometric Model

In general, a geometric model roughly divides the human body into several parts. Each part can be regarded as rigid and then fitted by the 3D geometric shapes, including general cylinder, ellipse, and rectangle. Knoop et al. [9] proposed a new method of fusing different input signals for human tracking. The algorithm can process 2D and 3D input data from different sensors (such as ToF camera, stereo or single-ocular images). For the tracking system, a 3D human model was built with several parts, in which each part was represented by a degenerate cylinder. The top view and bottom view of each cylinder can be regarded as an ellipse structure. Moreover, the two ellipses cannot rotate with each other and their planes were parallel. Therefore, a cylinder model needed to be described by five parameters: the major axes and minor axes of these two ellipses, together with the length of the cylinder. The entire 3D human model was composed of 10 cylinders, of which the torso started to extend as the root node. Each child node was described by a degenerate cylinder and the corresponding transformation of its parent node. 

The Head–Neck–Trunk (HNT) deformable template, represented by circles, trapezoids, rectangles, and trapezoids, was proposed in 2011 [10]. Once the HNT template works, the limbs (i.e., two arms and two legs) would be detected and fitted with rectangles. The end points of the rectangle were regarded as the joints of the human body, and its depth information was used to determine whether the human body was in a self-occlusion state. When self-occlusion occurred, the part segmentation of the human body was triggered, and then the segmented limbs were fitted separately. Inversely, the joints of the human body were directly obtained containing the contact points between the geometric shapes and the end points. Suau et al. [11] proposed a fast method to localize five joints of human body on the point cloud. In this method, the geometric deformation model established by the basic curve evolution theory and the level set method [12] was adopted to spread the topological structure of the human body. Additionally, the Narrow Band Level Set (NBLS) method [13] was also expanded to filter the 2.5D data according to its physical area. With the purpose of maintaining the connectivity on the depth surface to facilitate the extraction of topological features, the calculated NBLS map was filled, and finally the geodesic distance was used to quickly locate the five end points corresponding the five extreme joints. Lehment et al. [14] used an ellipsoid model for the upper-body with nine basic body modules, including left/right upper arms, lower arms, hands, head, torso and neck. Since the ellipsoid is a 3D equivalent ellipse, it is easy to be generated and controlled. Even in the case of having no clue about the color or texture, it can also find the nearest neighbor points with the input point cloud to calculate the similarity of the likelihood function.

Unlike the above method, Sigalas et al. [15] used 2D information to estimate the pose of the 3D torso. The initial face identification of the human in 2D image was used to segment the area of human body from the background. Based on the illumination, scale and pose invariant features on the 2D silhouette, the 2D silhouette was extracted from a 2D body, and then the curve analysis was performed to initially assume the area of the human shoulder. Meanwhile, the 3D information of the point cloud was meshed to estimate the 3D shoulder coordinates. The ellipsoid model was finally fitted with the torso area of the human body by least-squares optimization; a set of anthropometric standards were also applied to further refine the 3D torso pose.

**Figure 5:**
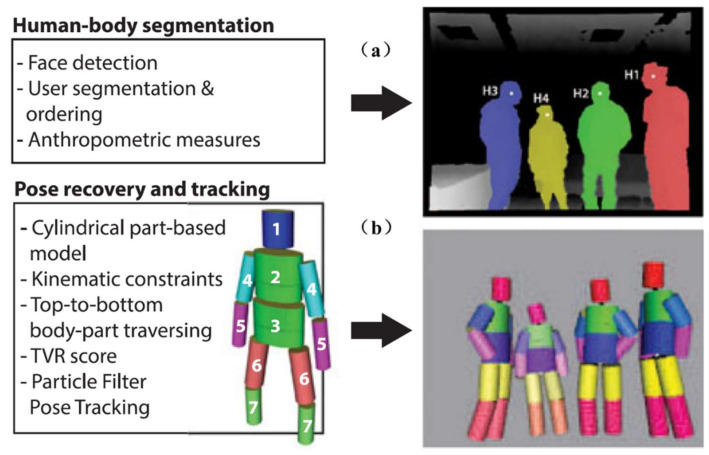
A geometric model can be applied to human joint estimation. (**a**) includes the human body segmentation and depth-based ordering and (**b**) includes the pose recovery and tracking. Figure from [16].

In order to increase the robustness of the algorithm, Sigalas et al. [16] further demonstrated a multi-person tracking system, as shown in Figure 5, in which human segmentation and pose tracking were contained. Human segmentation detected multiple human bodies by face detection in the depth map, and each human body was segmented individually. In the meantime, the length information of each part was calculated. Pose tracking first defined a body model with a head, upper and lower torsos, arms, and legs. Ellipse and circular were used to represent the upper and lower torsos, while cylinder could be implemented to fit for the remaining parts. Additionally, each part had a length limit. The point cloud of the human body obtained by the depth camera was rotated to the top view, and the reprojection ratio *f_reproj_* in Equation (1) was introduced as a matching index.

(1) $$f_{reproj}=\frac{N_{Pr}}{N_{3D}}$$

Multiple views by rotating the cylinder model around the *x*-axis can be generated, including occluded and non-occluded. For each view of the cylinder, the corresponding reprojection ratio visible points *N_Pr_* to the total number of 3D points *N_3D_* in the point cloud was calculated. Its value varied with the view, and it reached the minimum in the top view of the cylinder when the view-axis of the camera was aligned with the long axis of the cylinder. 

Based on T-pose, Wu et al. [17] created a simplified human skeleton model with customized parameters to adapt to different body types. The depth image and the corresponding 3D point cloud, as a pair of inputs, were first pre-processed and initialized to obtain personalized parameters. The torso part could be detected on the binarized image, the centroid of this part was calculated as the root node afterwards, and then using the root node as the parent node to iteratively find other child nodes. After obtaining the length between nodes, the human skeleton information was obtained by matching with skeleton model and further optimizing the joint angle. Besides, this method used the threshold segment to solve the self-occlusion problem.

#### 3.1.2. Mathematical Model

Mathematical model mainly transfers conceptual knowledge commonly used in mathematics to model construction. The basic idea is to build a model with the representation method of probability distribution to list each possible result and give their probabilities. Significant amount of work has been accomplished using Gaussian Mixture Model (GMM) in recent years. GMM is to establish a mixed model based on multiple Gaussian distributions for each pixel in the image. The parameters in the model are continuously updated according to the observed image, and background estimation is performed at the same time. Based on GMM, an algorithm [18] was proposed based on a single depth camera to estimate the pose and shape of the human body in real time. Due to the probabilistic measurement, it did not require explicit point correspondences. The articulated deformation model, which is based on exponential-maps, can direct embed into the GMM model. However, this algorithm simply used the first few frames to acquire human pose in the dynamic scenes, which usually did not provide the complete information. To cope with the time-varying articulated human body shape, Xu et al. [19] applied a GMM model to establish the pose and shape of the observed user. This method obtained the correspondence between the model and the user, and realized the shape estimation of human body based on multiple RGB-D sensors without any priori information. Compared with a single view case, depth data from multiple RGB-D sensors can not only handle more complex poses, especially occlusion situations, but also can be used to achieved different types of shape estimation by changing body attributes such as height, weight or other physical characteristics. Ge et al. [20] constructed a new non-rigid joints registration framework for human pose estimation by improving the two latest registration techniques. One is Coherent Point Drift (CPD), and the other is Articulated Iterative Closest Point (AICP). The GMM model was applied to initialize the standard pose of the human body through the CPD, and then AICP was employed with other pose point clouds to complete the pose estimation task. In the follow-up work, for incomplete data caused by self-occlusion and view changes, an effective pose tracking strategy was introduced to process continuous depth data [21,22]. Each new frame initialized a new template, which effectively reduced the ambiguity and uncertainty in the process of visible point extraction.

Stoll et al. [23] proposed Sums of spatial Gaussians (SoG) in 2015, which used a quad-tree to gather image pixels with similar color values into a larger square. It demonstrated remarkable performance for 2D data. Each square was represented by a Gaussian function, and then a set of isotropic Gaussian components constituted the SoG. Inspired by SoG, Ding et al. [24] presented Generalized SoG (G-SoG), which used an anisotropic Gaussian function with less calculation to represent the entire human body. On this basis, they expanded the 3D express form of SoG by grouping 3D parts of the point cloud with similar depth into voxels. The 3D Gaussian model only contained spatial statistical data, but not color information. 

Both SoG and G-SoG involve pose tracking of different characters. The former represents observed point cloud through effective octree division, and the latter embeds a quaternion-based articulated skeleton to create a standard human template model. A single un-normalized 3D Gaussian *G* can be expressed as Equation (2):

(2) $$G\left(x\right)\;=\exp\left(-\frac{\left\|x-\mu^{2}\right\|}{2\sigma^{2}}\right),$$

where *x* is 3D coordinates, *μ* and *σ*^2^ are the mean and the variance, respectively. SoG has the form as Equation (3),

(3) $$K\left(x\right)\;=\sum_{i=1}^{n}G_{i}\left(x\right)$$

A 3 × 3 covariance matrix is introduced into Equation (2) to replace the variance *σ*^2^, an anisotropic Gaussian in Equation (4) is obtained as,

(4) $$G\left(x\right)\;=exp(-\frac{1}{2}{\left(x-\mu \right)}^{T}\left[\begin{array}{ccc} C_{11} & C_{12} & C_{13}\\ C_{12} & C_{22} & C_{23}\\ C_{13} & C_{23} & C_{33} \end{array}\right]\left(x-\mu \right))$$

SoG was used to represent the point cloud of the human body, and then registered it with the G-SoG body template for human tracking. An Energy function in Equation (5), including similarity term, continuity term and visibility term, to describe the similarity between G-SoG and SoG [25],

(5) $$\hat{\theta }=arg\underset{\theta }{\min}\sum_{i\epsilon K_{m}}-E_{sim}^{\left(i\right)}\left(\theta \right)\cdot Vis\left(i\right)+\lambda_{con}E_{con}\left(\theta \right),$$

the first term emphasizes the similarity of the two models, *V_is_* gives the visible state of each Gaussian function, and the second term is added to smooth the pose estimation. In addition, the overlaps on the 2D projection plane of the Gaussian functions were used to judge whether there is occlusion.

**Figure 6:**
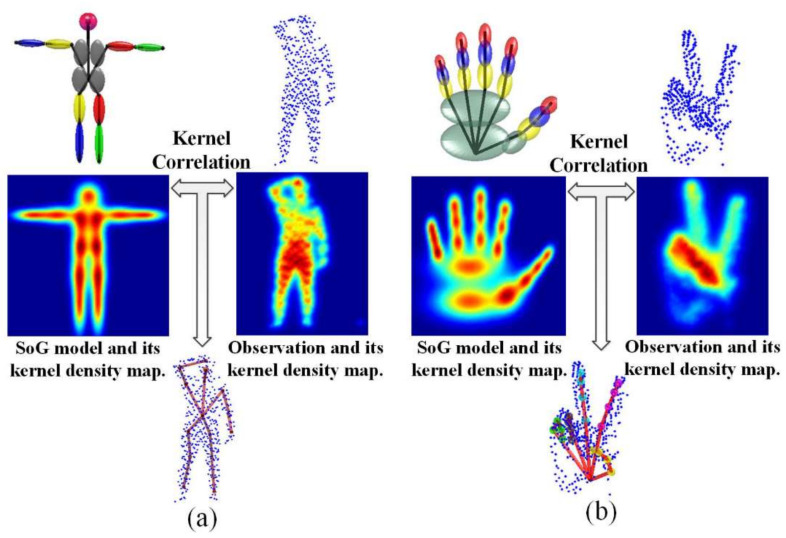
A conceptual scheme of mathematical model-based human joint estimation. Articulated pose estimation for the full body (**a**) and hand (**b**). The 1st row shows the Sums of spatial Gaussians (SoG) -based template models and an observed point cloud. Their corresponding Gaussian kernel density maps are depicted in the 2nd row, followed by the pose estimation results in the 3rd row. Figure from [26].

Based on the previous work, the author expanded the previous framework and proposed an articulated and generalized Gaussian kernel correlation (GKC)-based system [26], as shown in Figure 6, which supported subject-specific shape modeling and articulated pose estimation for the whole body and hands.

Apart from the method based on Gaussian distribution, Ganapathi et al. advanced a real-time tracking algorithm based on Maximum A Posteriori (MAP) inference in a probabilistic temporal model, and the human pose of each part was updated with Iterative Closest Point (ICP) algorithm. A two-stage method was proposed to solve the problem of recovering the human pose from a single depth map [27]. In the first stage, course template found in a large model dataset was used to make skeleton deformation, and then in the second stage, the detailed part of the human shape was restored by Stitched Puppet model [28] to fit the deformed model.

#### 3.1.3. Mesh Model

The mesh model is composed of many small polygonal patches in the computer to form the surface in the real world. Through the parameterized human body model, the structure has specific outer surfaces in addition to the skeleton, which reflects 3D appearance of the human body. Specific details such as characteristics are convenient to judge whether self-occlusion behavior occurs.

Ye et al. [29] built a fast pose detection system. After segmenting and denoising, the human point cloud was aligned with a series of mesh model, and the invisible parts were filled in the alignment process. Next, the shape and pose were deformed to perform fine-tuning. For the point cloud that failed to be registered, they searched the best alignment model again to complete the joint extraction process. Grest et al. [30] used the ICP algorithm with nonlinear optimization technology to achieve the purpose of aligning the mesh model and the human point cloud. By using the ICP algorithm, Park et al. [31] also recorded and processed multiple depth point clouds of single person from different perspectives to capture the shape of the entire body. Using template matching and Principal Component Analysis (PCA), a statistical body model representing a variety of human shapes and poses can be generated. PCA shortened the searching time by projecting the data into a low-dimensional Principal Component (PC) space. The ICP algorithm was adopted to fit the subject-specific human body model and depth data frame by frame, so that the accuracy of the original joint positions estimated by the Software Development Kit (SDK) was improved.

Hesse et al. [32] exerted a combination of texture model and random forest to classify body parts. According to the parts of human body, the position of the human joint was estimated. Vasileiadis et al. [33] used 3D Signed Distance Functions (SDF) data to represent the model, which was extended by a supplementary mechanism to track the pose of the human body in the depth sequence. In the actual multi-person interaction scene, the depth data of the human body in different perspectives was collected [34], and the mesh model was used for fitting to eliminate contact joint error. A new unsupervised framework was proposed to eliminate the influence of noise [35]. The method consisted of three steps: the deformed model and the human point cloud were registered with non-rigid point method to establish point correspondence, and the skeleton structure was extracted from the new point set sequence based on the cluster. Finally, Linear Blend Skinning (LBS)-based joint learning refine the positions. Huang et al. [36] estimated the joint positions by fitting a reference surface model, which included a reference triangle mesh surface and an inherent tree-shaped skeleton. Walsman et al. [37] utilized mesh templates to track human pose in real time and reconstructed high-resolution surface silhouette, so that it can facilitate gesture recognition and motion prediction using commercial depth sensors and GPU hardware.

Among all the mesh models, as a prominent and parametric human body model, Skinned Multi-Person Linear (SMPL) model can carry out arbitrary shape model and animation drive. This method can simulate the bulges and depressions of human muscles during limb movement. Therefore, the surface distortion of the human body during exercise can be avoided, and the appearance of human muscle stretching, and contraction can be accurately described. Zhou et al. [38] employed MobileNet to build a 2D human skeleton model, which facilitated the initialization of the point cloud. And then the customized SMPL model was fitted to the observed point cloud. The error was gradually reduced between the SMPL model and the actual observed point cloud by minimizing the loss function.

**Figure 7:**
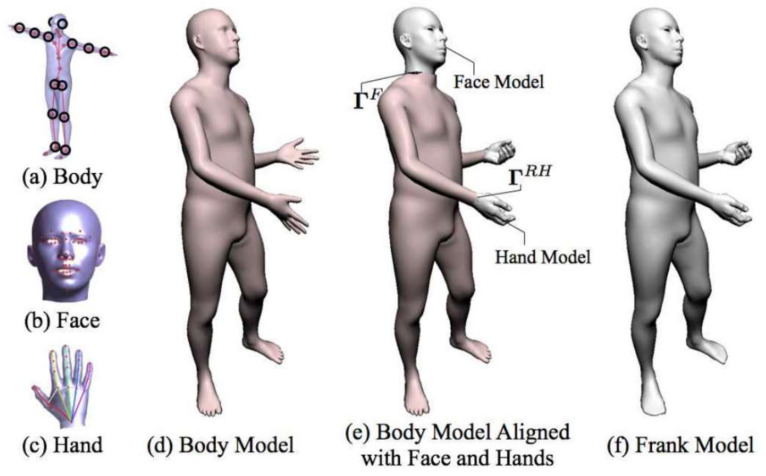
A mesh model is used to fit with point cloud of human body. (**a**) SMPL model; (**b**) FaceWarehouse model; and (**c**) artist-defined hand model. In (**a**–**c**), the red dots represent 3D positions of the corresponding keypoints reconstructed by detectors; (**d**) Body model; (**e**) Face and hand models are aligned with the corresponding parts of the body model; and (**f**) The whole Frank model. Figure from [39].

In order to enhance the generalization ability of the model, Joo et al. [39] designed a unified deformable model “Frank” to capture human motion at multiple scales without markers, including facial expressions, body motions and gestures. Figure 7 illustrates the main components of Frank. Each part is fitted with FaceWarehouse [40], SMPL model and artist-defined hand, respectively. Finally, the three models are partially spliced to capture human body motion and subtle facial expressions. The seamless mesh *V^U^* in Equation (6) was denoted as the motion and shape of the target subject. The main components of the Frank model *M^U^* include motion factor *θ^U^*, shape factor *φ^U^*, and global transformation factor *t^U^*.

(6) $$V^{U}=M^{U}\left(\theta^{U}+\varphi^{U}+t^{U}\right)$$

The motion parameters *θ^U^* in Equation (7) and shape parameters *φ^U^* in Equation (8) of the Frank model are the combination of each sub-model parameters,

(7) $$\theta^{U}=\;\left\{\theta^{B},\;\theta^{F},\;\theta^{LH},\;\theta^{RH}\right\},$$

(8) $$\varphi^{U}=\;\left\{\varphi^{B},\;\varphi^{F},\;\varphi^{LH},\;\varphi^{RH}\right\},$$

where *B* represents SMPL model, *F* belongs to face model, *LH* and *RH* are abbreviations of the left- and right-hand models, respectively. The motion parameter *θ^U^* mainly expresses the overall motion pose of the human body, including the relative angle information of the joints, and the shape parameter *φ^U^* is defined as the ratio of length, width and height.

The next step is to merge the model with the point cloud. There are two cases: one is when the corresponding point can be found between the model and the point cloud, the other is when the corresponding point is not obvious. In the first case, 2D detection first was operated to find the corresponding keypoints in each sub-region, and then converted them to 3D space. In the second case, the ICP algorithm was exerted to register the point cloud with model. The final objective function can be written as Equation (9):

(9) $$E\left(\theta^{U},\varphi^{U},t^{U}\right)\;=E_{keypoints}+E_{icp}+E_{seam}+E_{prior}$$

*E_keypoints_* means 3D keypoints detections, the term *E_icp_* expresses the cost of ICP algorithm. The skeleton hierarchy of the Frank model was closely connected. However, the independent surface parameterizations of some sub-model may lead to the introduction of discontinuities at the boundary. To avoid this artifact, the difference *E_seam_* between the vertices of the last two circles around the seam was minimize. Because the SMPL and FaceWarehouse model did not capture hair and clothes, the full body could not be explained well by the model. This resulted in incorrect registration during ICP. Hence, *E_prior_* was set on the model parameters to avoid overfitting the model to these noise sources. Furthermore, a new model, Adam, was derived to better capture the rough geometry of the human body with clothes and hair to match the geometry of the source data more accurately. 

This method above showed the potential that the unmarked motion being captured can eventually surpass the mark-based one. The marker-based method is very susceptible to occlusion, which makes it difficult to capture the details of the body and hands at the same time. This work can not only solve the occlusion problem, but also achieve higher precision model fitting results.

### 3.2. Feature-Based Methods

Global feature is a common feature-based method, which refers to the overall attributes. Common features include color, texture, and shape features. Because it is a low-level visual feature at the pixel level, the global feature possesses low variance, simple calculation, intuitive representation, etc. Among the global features, geodesic distance and geometric feature are commonly used in the point cloud-based applications.

#### 3.2.1. Geodesic Distance

Geodesic distance is literally inferred to be the shortest path distance between two points, which is different from the Euclidean distance that usually being used in the geometrical space. Euclidean distance is the shortest distance between two points in space, and geodetic distance is the shortest path of two points along the surface of the object. To solve the shortest path problem, Dijkstra’s algorithm [41] was commonly used, which was a greedy algorithm. This was because after specifying the starting point and ending point, the algorithm always tried to access the next node that is closest to the starting point in each step of the loop, thereby gradually obtaining the shortest distance between the two points.

Krejov et al. [42] located and separated the left and right hands according to the image domain, and then processed each hand in parallel to build a weighted graph on the surface. An effective Dijkstra’s algorithm is utilized to traverse the entire graph to find *N* candidate fingertips. With the shortest path algorithm, multi-touch interaction among multiple users is realized. Phan et al. [43] proposed an online multi-view voting scheme (MVS) running at an interactive rate. It combined the measurement results from multiple sources to generate a fine geodesic distance graph (GDG), and then five geodesic extremes in the GDG were marked as the head, hands, and feet. Assuming that the length of each bone is determined in advance, so additional landmarks are obtained by calculating the centroid of each region, corresponding to the secondary joints of the wrist, elbow, knee, ankle, and neck. To overcome the errors caused by misdetection and occlusion, an improved method using feature point trajectories to correct the error detection was designed [44]. Five extreme points were detected by geodesic distance method. A shoulders template was applied to search for the position of the shoulders. Once the shoulder joint is determined, the geometric midpoint was regarded as the position of the elbow joints. An iterative search method was used to find the elbow point by minimizing the total geodesic distance from the shoulder point to the hand point through the elbow point. Besides, a minimum distance constraint was imposed afterward in the corresponding recognition to predict its most likely spatial position in the next frame for tracking the trajectory of each joint. To solve the problem of detecting and identifying body parts in the depth data at the video frame rate, a solution was proposed to obtain a new interest point detector on the point cloud data [45]. First, the extreme points were detected by using the geodesic distance, and were further divided into hands, feet, or head using local shape descriptors, and 3D direction vector of each point is given. To speed up the search process of candidate points in the human body, a quadtree-based method was utilized to effectively group adjacent data, and then Dijkstra’s algorithm was applied on this basis to obtain the feature points. In the tracking process, a noise removal and restoration method based on Kalman filter was used to correct and predict the extreme positions [46].

Combining multiple methods can also provide better accuracy in estimating the position and direction of the joints. Handrich et al. [47] replaced depth information with more complex features describing local geodesic neighborhoods, and then a random forest classifier was used to learn the correct body part from these descriptors. Baak et al. [48] employed the geodesic distance, which was extracted from the input data as a sparse feature to retrieve the pose from a large 3D pose dataset, and merged it with the previous pose to achieve pose tracking. Mohsin et al. [49] described a system for successfully locating specific body parts. Multiple depth sensors were used to collect point clouds from different perspectives to help solve the occlusion problem. In order to locate prominent human limbs, a triangular mesh model was applied to the 3D point cloud, and the ends of the limbs were marked with geodesic distance.

**Figure 8:**
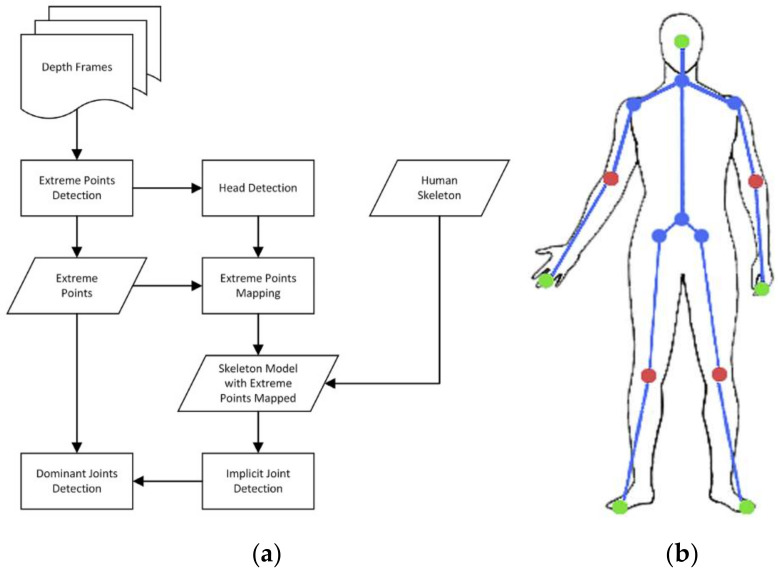
Geodesic distance is used to locate the positions of extreme points. (**a**) describes the overview of the workflow of the proposed method. (**b**) shows the skeleton model used in our method. The green dots represent the extreme points. Blue dots represent implicit joints (neck, waist, shoulders, and hips). Red dots represent dominant joints (elbows and knees). Figure from [50].

In general, the geodesic distance can only be used to detect the five extreme points of the human body, namely, the head, hands, and feet. A hybrid framework using depth camera to automatically detect joints was proposed [50]. This method divided the joints into two types: implicit joints and dominant joints. Dominant joints include extreme points, elbows, and knees. Implicit joints are points on the trunk, such as the neck and shoulders. The specific extraction process is shown in Figure 8a. Due to the rigidity of the human limbs, the dominant joints are easier to be detected than the implicit ones. First, the geodesic features of the human body are used to establish extreme points.

(10) $$D_{g}\left(p_{0},P\left(x_{p},y_{p}\right)\right)\;=\sum D_{g}\left(P\left(x_{p},y_{p}\right),P\left(x_{q},y_{q}\right)\right)$$

In Equation (10), *P* denotes the point cloud, *p_0_* is starting point, and *D_g_ (∙)* represents the geodesic distance between two random points. If the corresponding relationship between the extreme points and the skeleton model is not given, it is difficult to detect the position of the joints. Therefore, starting from mapping an extreme point to the head, the feature of the area around each extreme point is used to compare with the head model. Each extreme point is gradually mapped to the corresponding part of the human body model. In the skeletal model as described in Figure 8b, the geodesic distance between the head and the hand is smaller than the geodesic distance between the head and the foot, which is the criterion used to separate the hand and the foot joints.

With the above restrictions, the extreme points are found. The human skeleton model is then used to define implicit joints. Assuming that the geodesic distance between the left hand and the left shoulder is shorter than the geodesic distance between the left hand and the right shoulder. The relationship between the left hand and the right hand can be described as Equations (11) and (12):

(11) $$D_{g}\left(p_{Lh},j_{Ls}\right)\;<\;D_{g}\left(p_{Lh},j_{Rs}\right),$$

(12) $$D_{g}\left(p_{Rh},j_{Rs}\right)\;<\;D_{g}\left(p_{Rh},j_{Ls}\right)$$

By adding constraints such as Euler angle and geodesic distance ratio, the joint candidates were ensured to show the degree of curvature of the path. The strategy based on the global shortest path was adopted to detect the dominant joint candidates, such as elbow and knee joint, and then the shortest paths for specific detection were further used to locate these joint. Furthermore, to deal with self-occlusion, when the distance map is updated, the difference in depth values is calculated between adjacent points. If the difference is less than the threshold, the two points are on the same surface of the human body. Otherwise, they are in different parts of the human body.

#### 3.2.2. Geometric Feature

Geometric features refer to the overall attributes, common ones include texture and shape features of the human body. To eliminate the influence of complex poses by constructing and merging 3D point clouds of multiple views. The part detector was used to detect the body parts [51], and then the centroid of each part was obtained as the joint position. Based on the shape segmentation and skeleton sequence, Zhang et al. [52] designed an extraction method of human skeleton. In the preliminary step, the centroid of every part was also used to generate a pseudo skeleton. Multiple depth sensors were also utilized to achieve the purpose of motion capture [53]. First, multi-frame depth data from the depth sensor was converted into multiple point clouds, and then, these point clouds were combined into a merged point cloud, on which the skeleton line was acquired by the Reeb graph. Finally, the joint position was calculated from the skeleton line according to the joint structure of the human body. A curve skeleton expression based on the set of cross-section centroids was presented [54]. Patil et al. [55] applied multiple inertial measurement unit (IMU) sensors, which were placed at the human joints to estimate the 3D position of the joints, the Lidar data compensate for displacement drift during the initial calibration of the skeleton structure. A 2.5D thinning algorithm was exerted [56], including segmentation of the occlusion region and thinning line extraction. The thinning line bone obtained cannot determine the exact position of all body joints, but the end-joints of the body part can be detected. Finally, it was registered with the constructed human model containing 16 bone joints, and the human joints were extracted.

**Figure 9:**
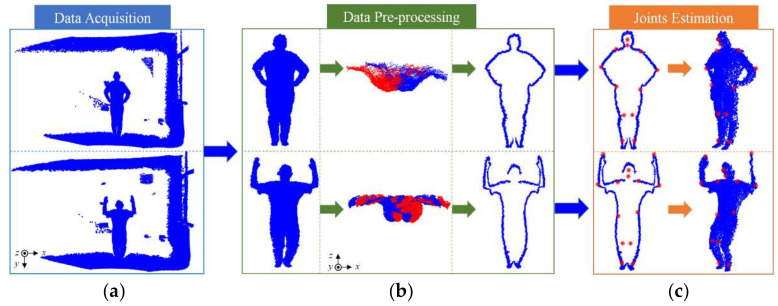
Geometric feature is used for human joint estimation. The approach consists of three stages: data acquisition, data pre-processing and joint estimation. (**a**) The point clouds are directly obtained from the depth camera. (**b**) Data pre-proposing mainly involves three parts: firstly, the irrelevant points are filtered, then the orientation of the human point cloud is adjusted, and finally the 3D silhouette is extracted. (**c**) Fourteen joints of human body are extracted by using the geometric feature of human silhouette. Figure from [57].

Xu et al. [57] detected the human joints in a single-frame point cloud using the TOF depth camera. The process was distributed into three stages as shown in Figure 9. An in-house captured 3D dataset containing 1200-frame depth images was first collected, which can be categorized into four different poses (upright, raising hands, parallel arms, and akimbo). To eliminate the influence of the background and noise points on the algorithm, the point cloud was separated from the background by the conditional filtering in the data pre-processing stage. To avoid self-occlusion, the point cloud was projected to the 2D top view, and then the point cloud was easily rotated by the angle, which was formed by the farthest points on the *x*-axis and the horizontal axis, to make the viewpoint of the camera parallel with the direction of the human body being facing. Finally, the 3D silhouette of the human body was extracted by adopting the public algorithm in the PCL [7] as a global feature in the point cloud.

Before extracting the joints of human body, different poses were classified according to the angle and aspect ratio of the silhouette point cloud. First, the four poses are divided into two categories, according to the angle formed by the farthest point on the *x*-axis and the point with minimum value on the *y*-axis, one includes upright and akimbo poses, and the other contains the remaining poses. Then, the two poses in each category are further distinguished according to the aspect ratio of the silhouette. There was slight difference in the extraction method of the 14 joints for different poses. The approximate flow was that the head and foot joints were regarded as the centroids of each segmented part according to body proportion. As the base point, the waist joint was obtained in the next step using the prior information, while the shoulder and hand joints can be acquired afterwards. The elbow and knee joints were calculated by judging whether bending was present. In an upright state, the elbow joint was determined to be the midpoint between the hand joint and the shoulder joint, while the knee joint was also located on the line between the foot point and the midpoint of the left and right shoulders. When in the bent state, the elbow joint was defined the farthest point in the arm point cloud from the straight line formed the hand and shoulder joints, and the knee joint was the minimum value in the *z* direction.

Compared with the other methods, the accuracy of the joints was greatly improved. The average joint error was less than 5.8 cm by using both the in-house and public datasets, but it was also affected by the clothes, which led to more error in the waist joint.

### 3.3. Machine Learning-Based Methods

Given the rapid development of machine learning technology in computer vision, some of the latest deep learning networks, such as PointNet [58], VoxelNet [59], PointCNN [60] and PointConv [61], are also implemented in the 3D point clouds. These algorithms have further pushed the development of deep learning on 3D point clouds to address various problems [62,63]. This review attempts to track and summarize the progress of point cloud-based networks for human tracking in recent years, so as to provide a clear prospect for the current point cloud-based joint extraction of the human body. We mainly summarize from two categories of neural network and classification tree.

#### 3.3.1. Neural Network

**Figure 10:**
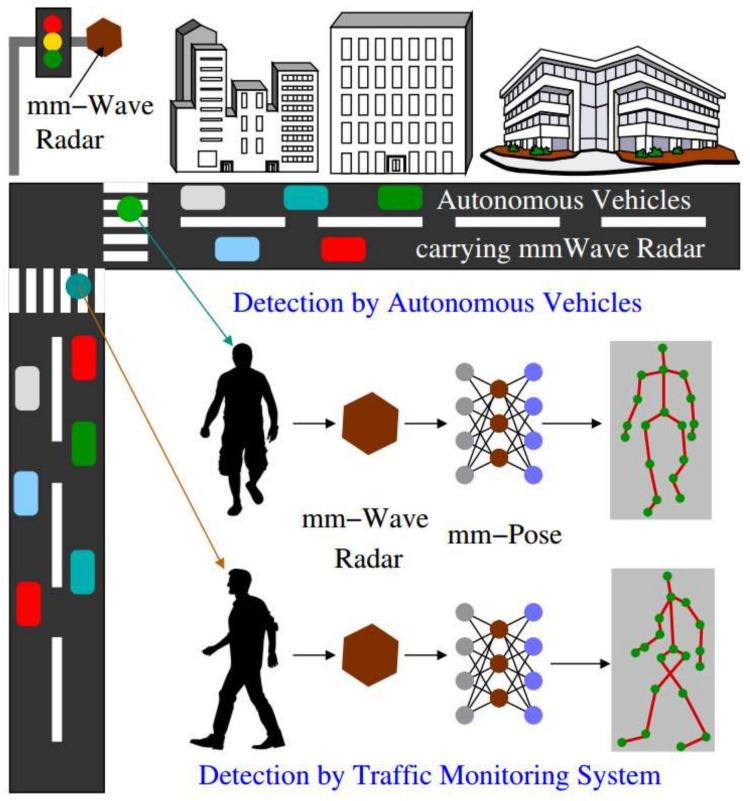
In automatic/semi-autonomous vehicles and traffic monitoring systems, mm-Pose can be used to perform robust skeleton pose estimation of pedestrians. Figure from [69].

One very important field of machine learning is the neural networks. Especially, convolutional neural network (CNN) is a fascinating and powerful tool that can achieve great analysis results in many tasks of computer vision. A 2D CNN is used to locate 2D human joints, which are then extended to 3D through a depth transformation to reduce the computational cost. Biswas et al. [64] designed an end-to-end system that combines RGB images and point cloud information to recover 3D human pose. Özbay et al. [65] used a simplified extraction method “Conditional Random Field” to classify 3D human point clouds, and the corresponding images and poses as input of CNN transmitted similar spaces. When the image-pose pair is matched, the value of dot product is high, otherwise the value is low. Without making any assumption about the appearance and initial pose of the human, the proposed system could be applied to multi-human interaction scenarios [66]. Schnürer et al. [67] utilized networks to generate 2D belief maps, combined with depth information for pose detection of the upper body, which required fewer resources while achieving a high frame rate. However, the depth mapping of a 2D single-channel image did not represent an actual 3D representation. To overcome this limitation, a 3D CNN architecture was proposed to provide a likelihood map for each joint [68], and the detection structure was extended to make it suitable for multi-person pose estimation. Millimeter wave (mmWave) has the advantages of high bandwidth and fast speed, which is the reason why it is used as the carrier of 5G technology. A new method of real-time detection and tracking of human joints using mmWave radar was proposed [69], named mmPose. This is the first method to detect different joints using mmWave radar reflected signals, and the emission wave at 77 GHz allowed it to capture small differences from the reflective surface. The algorithm structure is shown in Figure 10.

The objects reflected the radar signal within a coherent processing interval (CPI), and a 3D radar cube was obtained with fast-time, slow-time and channel. In order to overcome the sparseness of the voxel grids. and significantly reduce the subsequent machine learning structure, the depth, the ratio between elevation and azimuth, and normalized power values of the reflected signal were assigned to the RGB channels to generate a 3D heat map, which can be used as the input to CNN, and the output of CNN were different human joints in 3D space.

**Figure 11:**
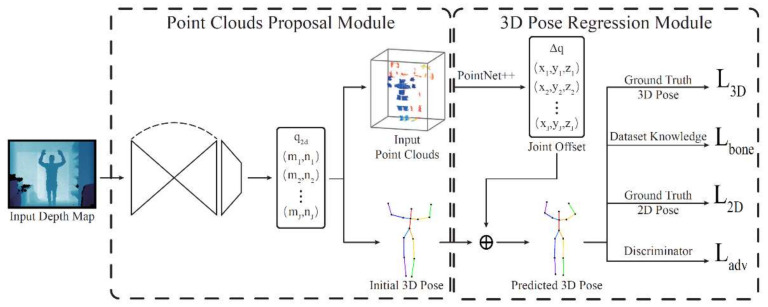
A schematic diagram of human joint estimation using Neural Network. The network consists of two modules, the point clouds proposal module and the 3D pose regression module. Using the input depth map, we first estimate the 2D human pose, and use it to sample and normalize the extracted point clouds from depth. Then we use the initial 3D pose converted from the estimated 2D pose and the normalized point clouds to predict the final 3D human pose. Figure from [63].

In addition to CNN, other neural networks are also commonly used in point cloud-based pose recognition. Fully connected network (FCN) was introduced to accurately simulate the restriction of the human joint [70], which can effectively implement the realistic restriction by transforming the constraint force in the physics engine into an optimization problem. Li et al. [71] proposed multi-layer residual network to obtain hand features for tracking and segmenting. Zhang et al. [63] adopted an adversarial learning method to ensure the effectiveness of the restored human pose to alleviate the ambiguity of the human pose caused by weak supervision. A deep learning-based weakly supervised network, as shown in Figure 11, not only used the weakly-supervised annotations of 2D joints, but also applied the fully supervised annotations of 3D joints. It is worth noting that 2D joints of human body can help select effective sampling points to reduce the computational cost of the point cloud-based network.

In this paper, a point cloud-based network is involved. Initially, Qi et al. [58] proposed the PointNet network, which can extract features of point from unordered point clouds. PointNet used traditional multilayer perceptrons (MLPs) as the core learning layer. It is commonly used to deal with 3D object classification and point-level semantic tasks. In the subsequent research work, PointNet++ [72] added local structures at different scales to enhance PointNet. Because of the effectiveness of this method, the author used the PointNet++ network to deal with point segmentation. Compared with the existing methods about pose estimation of the human body that require human foreground detection, this method can perform accurate pose estimation without clear requirements. 

Joint extraction of part structures from human body attracted much attention for further research. The proposed self-organizing network aims to use unannotated data to obtain accurate 3D hand pose estimation [73]. The heat map, as the output of 3D CNN, reflected the probability distribution of the joints. In [74], heat map was used as the intermediate supervision of the 3D hourglass network to participate in the skeletal constraints for the hand tracking. In addition to the heat map representing the distance, the unit vector field was introduced, and joint position was inferred by weighted fusion [75]. In order to further improve the accuracy of the fingertips, a fingertip refinement network was designed to model the visible surface of the hand and perform pose regression [76]. Different from the original PointNet, Local Continuous PointNet (LCPN) [77] was proposed to extract the local features of the neighbor index in the unorganized point cloud to estimate the facial joints. The input of the 3D CNN was encoded through the projection of the point cloud [78]. After the convolution and pooling layer, 3D features can be extracted from the volume representation, which can be used to return the relative position of the hand joints in the 3D volume. An end-to-end multi-person 3D network Point R-CNN for the pose estimation was proposed [79], which used panoramic point clouds of multiple cameras to solve the occlusion problem. The whole network can be regarded as a combination of two parts. The first segment is for the instance detection using VoxelNet and the other segment is for instance processing by the PointNet to acquire the joint information.

#### 3.3.2. Classification Tree

The classification tree is one of the prevailing methods for human body segmentation. As a newly emerging and highly flexible machine learning algorithm, random forest (RF) refers to a classifier that uses multiple trees to train and predict samples, has a wide range of applications.

Inspired by the decision forest, each point in the point cloud of the human body is voted to evaluate the contribution of each part of the human body, so that collaborative method was proposed to learn the 3D features of the human body [80]. Xia et al. [81] trained the cascade regression network from the pre-recorded human motion dataset. In addition, the hierarchical kinematics model of the human pose was introduced into the learning process, it can directly estimate the accurate angles of the 3D joints.

Random forest is often used to segment different parts of the human body in the following literatures. Different regions of the upper body were first detected, and then the probability map for each region were calculated [82]. The highest part in the probability map was defined as the external joints. The internal joints, such as the elbow, were fitted with an ellipse model to obtain. In the 3D point cloud, the Principal Direction Analysis (PDA) was used to estimate the main direction of the body part, and then the main direction was mapped to each part of the 3D model to estimate the human pose [83]. In the prescribed action set, a pose estimation using multiple random forests was proposed to enhance the results of motion analysis [84]. A group of random verification forests were set to verify classification results of the initial random regression forest for precise joints positioning. The geodesic-based feature descriptors played a significant role in the random forest classifier to produce more exact spatial predictions for body parts and bone joints [85]. Random forest was also applied to infer the consistency between the input data and the construction template [86]. The method successfully restores the shape of the human body and extracts joints.

The method of pixel inference using random decision tree usually requires more heavy calculation. Especially when the number of trees is increased to improve generalization and accuracy, the computational burden of multiple trees may force a trade-off between speed and accuracy, and the random tree walk (RTW) method can obtain greater gain. The method combined RTW with optimization methods such as ICP and random search, which raised the ability to extend of the classification tree [87]. RTW was used to initialize various assumptions in different ways and then passed them to the optimization stage.

Yub et al. [88] no longer trained the tree for pixel-level classification, and used the regression tree to estimate the probability distribution towards a specific joint direction relative to the current position. In the test process, the direction of random walking was randomly selected from a set of representative directions. A new position by a constant step was found in that direction.

**Figure 12:**
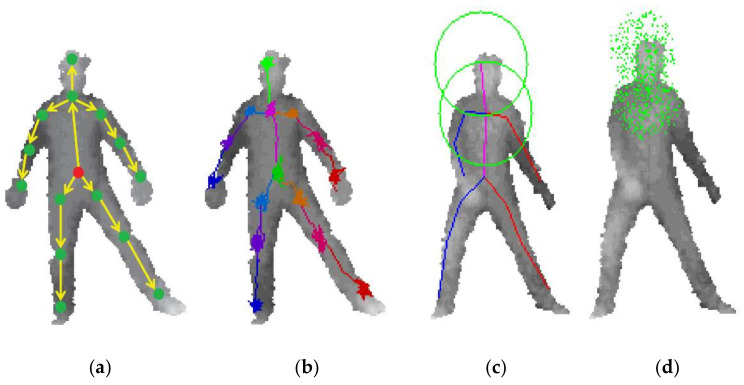
Example of classification tree for human joint estimation. (**a**) illustrates the kinematic tree implemented along with random tree walk (RTW). First, the random walk toward belly positions starts from body center. The belly positions (red dot in (**a**)) become starting point for hips and chest, and so forth. (**b**) shows the RTW path examples. (**c**) illustrates offset sample range spheres in green. In (**d**), the green dots represent offset samples. Figure from [88].

For all positioning problems, as long as we know the direction of any point on the object towards that position, we can find the correct position. Ideally, the orientation of all parts should be trained from all possible positions of the whole body, because random tree walking could reach the joint position faster, so a starting point close to the target joint position was required. In the case of using the skeleton topology, one needed to provide a nearby initial point for the RTW, as shown in Figure 12.

RTW can be described as training regression trees for each joint in the human skeleton. Here, the direction from the point to that specific joint is obtained by training a regression tree. Therefore, a training set is first constructed with the position of each joint point and the depth value of the input point.

The unit direction vector $\hat{u}$ from the offset point to the joint was defined as Equation (13):

(13) $$\hat{u}=\;\left(p_{j}-q\right)/\|p_{j}-q\|,$$

where $p_{j}$ is the coordinate of a random point, *q* is the position of the specific joint. The training sample *S* is expressed as Equation (14):

(14) $$S=\;\left(I,q,\hat{u}\right),$$

*I* represents the depth value. The goal is to find a partitioned binary tree that minimizes the sum of squared differences. At the same time, the directions are stored on each leaf node in the form of clusters, so that several representative directions and corresponding probability weights form the output of the tree. When estimating the pose, the path starts walking randomly from some initial points. In each step of the traversal, the regression tree is traversed to a leaf node, where a set of directions corresponding to the current point can be obtained. However, the step direction is randomly selected from the k-means cluster unit vector at the leaf node.

### 3.4. Summary

The template-based method first needs to establish a template library or a parameterized template, and then the similarity between the point cloud of human body and the sample in the template library or the target model is compared. This method is relatively rough and time-consuming. Given diversity and multi-scale structure of the sample data, the same pose of human body may be very different in space. Therefore, the accuracy of template-based methods is very limited. 

Feature-based method needs to extract the global or local features of the point cloud, which combine with some prior knowledge to obtain the 3D joints of the human body. This method relies on the selection of feature points such that it is not suitable for self-occlusion and changing poses. Therefore, it is necessary to further optimize the robustness of the algorithm for covering the poses of the human body as much as possible. 

The machine learning-based method mainly uses the network to automatically learn the required features from the point cloud, and then the learned features can be regarded as the judgment condition to extract the human joints. Compared with the above two methods, it has been greatly improved. On one hand, the obtained joints can achieve higher accuracy by learning sample features in a large training set, and on the other hand, it is also very robust to scale processing. The machine learning-based method can make up for the shortcomings of the above two methods, but it is restricted by the sample richness of the training set, so the construction of the training set is very important to the machine learning-based method. 

Moreover, we summarize some works of point cloud-based joint estimation for human body in Table 2. For 3D pose estimation, two different error metrics can estimate the accuracy of the method. One is direct measurement of the Euclidean distance between the estimated and ground truth joints, another is the average precision (AP), which is defined as the ratio of correctly estimated joints within a specific threshold. Some works have adopted the AP error metrics, and Table 2 reports datasets together with some key parameters, especially, the threshold values *δ.* When *δ* changes, the AP of each joint would be different. The 3D coordinate of each classified joints, which are obtained by proposing algorithm in the literature, is compared with the corresponding ground truths of the joints within the same dataset. When the difference between the two values above is less than the given threshold *δ* in Table 2, the joint coordinate is considered to be a correct position. For acquiring the tracking accuracy of each classified joint in the referenced works, the ratio AP in Table 3 is finally calculated between the sum of the correct locating joints and all joints.

## 4. Public Depth Dataset of the Human Body

Public datasets play an important role in testing the robustness of the algorithm and provide a platform to compare different algorithms in a fair manner. In the past few years, many 3D benchmark datasets for different applications have been collected and made publicly available to the research community. The structure of the dataset mainly includes RGB, depth map or point cloud acquired from structured light or ToF depth camera. In this paper, we only focus on the point cloud-based dataset. This section provides a detailed review of the datasets listed in Table 4. Since point cloud occupies fairly large storage space, most datasets usually provide depth maps together with the internal parameters of the camera, which can be easily converted to the point cloud-based datasets.

A set of widely availed depth dataset, named SMMC-10, was constructed as a benchmark for the algorithm testing [90]. To generate this dataset, a probability model containing 15 rigid parts of the human body was first defined. These rigid parts were spatially constrained by the joints with 48 degrees of freedom. This dataset was recorded by the Motion Capture (MoCap) system and the ToF camera (Swissranger SR4000) at 100–250 ms per frame. It included 28 real actions, such as fast kicking, swinging, self-focusing, and whole-body rotation.

Another constructed dataset by Li et al. [91], named MSR-Action3D (https://documents.uow.edu.au/~wanqing/#MSRAction3DDatasets (accessed on 12 June 2020)), was 20 game action for seven subjects facing the depth camera, including: high arm wave, horizontal arm wave, hammer, hand catch, forward punch, high throw, draw x, draw tick, draw circle, hand clap, two hand wave, side-boxing, bend, forward kick, side kick, jogging, tennis swing, tennis serve, golf swing, and pickup and throw. Each action was captured three times by Kinect v1 (Microsoft Corp., Redmond, WA, USA) at 15 frames per second. In total, the dataset reasonably covered various movements of arms, legs, and torso, which stored 4020 motion samples with 23,797 depth maps. Notes that, if an action was done with only one arm or one leg, subjects were advised to use their right arms or right legs. 

A large-scale RGB+D human action recognition dataset, named NTU RGB+D dataset (http://rose1.ntu.edu.sg/Datasets/actionRecognition.asp (accessed on 12 December 2020)), used a recurrent neural network (RNN) to simulate the long-term time correlation of various parts in the human body, and to better classify the human body poses [92]. The dataset collected more than 56,000 video samples with a total of four million frames from 40 different subjects and 60 different operation classes, including daily operations, interoperations, and health-related operations. 

Nguyen et al. [93] explored the extraction of skeleton during human walking. The content of the walking gait dataset was about 18.6 GB, and it was divided into a test set and a training set. The test set included samples of five subjects, and the training set was walking gait data of four individuals. The data collected for each person includes the information of skeleton and silhouette together with the point cloud.

Berkeley Multimodal Human Action Database (MHAD) took seven males and five females aged between 23 to 30 years old as subjects (http://tele-immersion.citris-uc.org/berkeley_mhad (accessed on 2 November 2020)) [94]. Each subject performed 11 actions in succession, such as jumping, throwing, waving, sitting down and so on. To ensure the accuracy of action acquisition, all subjects repeated 5 times for each action, resulting in a total of approximately 660 action sequences. In addition, a T-pose model was created for each subject to extract its corresponding skeleton.

The G3D dataset (http://dipersec.king.ac.uk/G3D/G3D.html (accessed on 29 June 2020)) mainly includes different game actions [95]. Given the internal parameters of the depth camera, the captured depth map can be converted into a point cloud. The dataset contains 10 subjects. Each subject was required to complete 7 action sequences consisting of 20 game actions: punch right, punch left, kick right, kick left, defend, golf swing, tennis swing forehand, tennis swing backhand, tennis serve, throw bowling ball, aim and fire gun, walk, run, jump, climb, crouch, steer a car, wave, flap and clap. On this basis, G3Di (http://dipersec.king.ac.uk/G3D/G3Di.html (accessed on 29 June 2020)) [96] was constructed to process a human interaction for multiplayer games. The dataset contained six pairs of subjects’ motion interaction behaviors, such as boxing, volleyball, football, table tennis, sprinting and hurdles. Each action was separately stored as RGB, depth and skeleton data. 

A complex human activity dataset, called SBU-Kinect-Interaction (https://www3.cs.stonybrook.edu/~kyun/research/kinect_interaction/index.html (accessed on 19 December 2020)), was created to describe the interaction between two people [97], including synchronized video, depth and motion capture data. All videos were recorded in the same laboratory environment. Seven participants performed the activity consisting of 21 groups, where each group contained a pair of different people performing all eight interactions. Note that in most interactions, one person was acting and the other was reacting. Each action category contained one or two sequences. There were approximately 300 interactions in the entire dataset.

The CDC4CV pose dataset [98] was acquired with the depth information of the upper body for comparison of static pose estimation techniques by the Kinect v1, including 9 joints of three subjects. During the acquirement of the depth pose of human body, the upper body of each subject was ensured to stay within the 640 × 480 window. Nearly 700 depth data including three subjects were collected and labelled, of which 345 depth data were chosen as the training set and the rest data were used as the test set.

The EVAL dataset was built in 2012 (http://ai.stanford.edu/~varung/eccv12 (accessed on 16 September 2020)) [89], which included 24 action sequences of three different subjects. Each subject performed actions of gradually increasing complexity at the place where is approximately 3 m away from a Kinect camera. The ground truth of the 12 joints was captured using the Vicon motion capture system, and stored in the EVAL dataset together with the corresponding 3D point clouds.

CMU MoCap (http://mocap.cs.cmu.edu (accessed on 14 October 2020)) [99] used 12 Vicon infrared MX-40 cameras to collect the motions of the human body wearing black jumpsuits, including six major categories, such as human interaction, interaction with environment, locomotion, physical activities and sports, situations and scenarios, and test motions. And each category was further divided into 23 sub-categories. 41 marks were posted on the human body for the cameras to collect the ground truth of the joints during the motion. The images captured by various cameras were then triangulated to obtain 3D data.

In summary, MoCap is a motion capture system by posting marks on the joints of the human body with multiple cameras to track the human joints from different views. Accurate 3D skeleton information at a very high frame rate is acquired in the system. However, the system is usually expensive, and only available in an indoor environment. At present, many methods also use a single depth camera for data acquiring and processing. The subjects do not need to wear any equipment with constraints. For the single person datasets, MSR-Action3D and G3D used gaming action as the main application. Both of them were single view and in the similar action sequence. In addition to depth data, MSR-Action3D also collected the video data, and G3D provided the corresponding RGB images at the relatively high frame rate. SMMC-10, Walking gait, MHAD, CDC4CNV and EVAL are mainly included basic behaviors of the human body. Only single individual was used to complete a series of complex actions in SMMC-10. MHAD contained the depth information of 12 subjects from four different views; the gender and age of the subjects were also given. EVAL provided the ground truth of 12 joints and the corresponding information of the 3D point cloud. CDC4CNV tracked the nine joints of the upper body, and Walking gait was used to analyze human gait, both of their application scenarios have certain limitations. For the multi-person datasets, the MoCap system can be used to obtain the ground truths of 41 joints in CMU MoCap dataset, it covered multi-person gaming, sport and other behaviors. SBU-Kinect-Interaction provided eight classes of interaction sequences. After that, G3Di provided common interaction activities for multi-person gaming, 20 joints of the human body were given for detailed analysis. NTU RGB+D used Kinect v2 to acquire the ground truths of 25 joints with more human interaction activities. In addition to the depth information, the dataset also included RGB images and IR videos.

## 5. Application of Point Cloud-Based Joint Estimation

**Figure 13:**
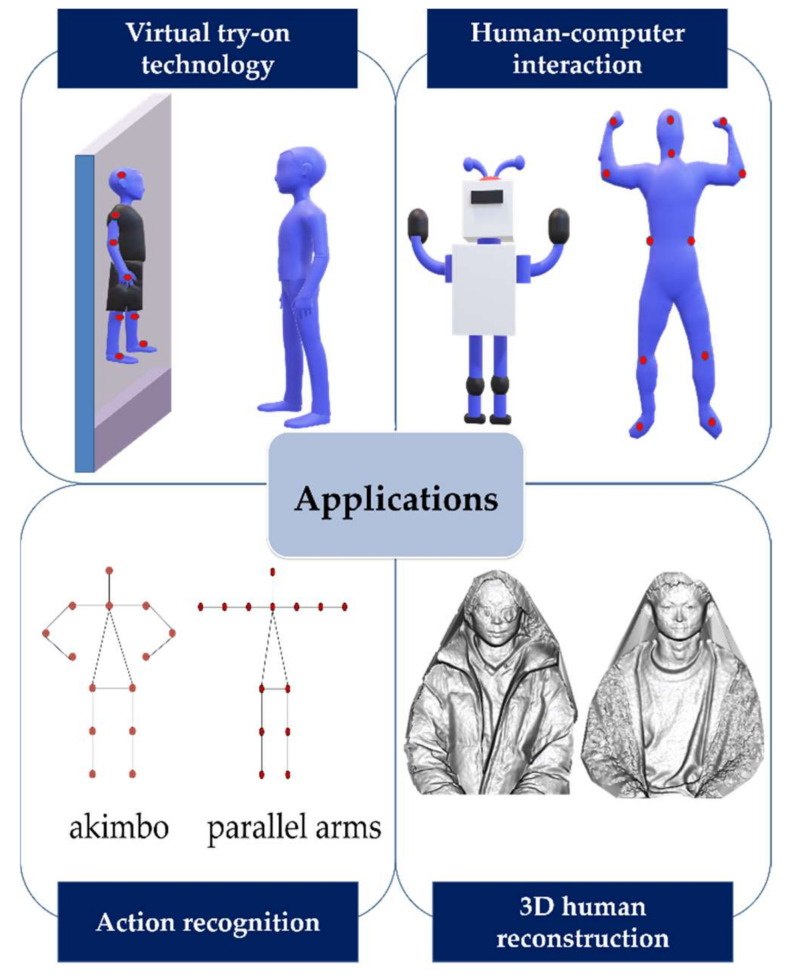
Applications of point cloud-based joint estimation.

Human joint recognition is one of the important directions of artificial intelligence applications. With the maturity of technology, human-related research can use joint information to solve some problems. According to different application scenarios, the approaches can be divided into the following categories, including virtual try-on technology, 3D human reconstruction, action recognition, human–computer interaction, and many others, some examples of the above application are shown in Figure 13. The related literatures are summarized according to the above application scenarios.

Human body shape estimation is essential for virtual try-on technology. Estimating the 3D human shape in motion from a set of unstructured 3D point clouds is a very challenging task. Human joints can play an important role as a priori in 3D shape estimation of human body. Yang et al. [100] proposed an automatic method to solve the estimation of the human body shape in motion. Under the premise of wearing loose clothes, the model reconstruction problem is expressed as an optimization problem by controlling the body shape. Based on the automatic detection of human joints, the pose fitting scheme was optimized [101]. The results of 3D scanning from multiple viewpoints were projected onto 2D images, and then deep learning algorithms were utilized to mark the joints, which were helpful to find the best pose parameters. With the help of the joints in the SMPL model and manually marked the joints in the point cloud for registration, the result of coarse registration was obtained, and the hot core feature was extracted between the two frames during the changing pose for non-rigid registration. Both the result of non-rigid registration and coarse registration was fitted each other to get the final 3D human body model [102]. The joints of the frontal point cloud of the human body, which was generated directly with Kinect device, helped initialize the personalized SMPL model, and the model was registered with the input point cloud to find the corresponding points for obtaining a 3D human body model [103]. Yao et al. [104] further projected the obtained model onto the corresponding RGB image.

The joints can also assist the 3D reconstruction of the human body. Matteo et al. [1] utilized the information provided by the skeletal tracking algorithm to transform each point cloud into a standard pose in real time, and then registered each transformed point cloud to achieve 3D human body reconstruction. In order to extract more point features, a graph aggregation module was used to enhance PointNet++ [2], an attention module was used to better map disordered point features into ordered skeleton joint, and a skeleton graph module aimed to regress the skeleton joints by SMPL parameters. A dataset containing 2D scenes and 3D human body models was constructed. After marking the joints of human body on 2D image, they were converted to 3D coordinate system generated by the radar, and the SMPL model was used to fit the pose of the human body [105].

To improve the accuracy and real-time performance of action recognition, the skeleton-based method is studied in various research fields as an effective technology. Instead of using the entire skeleton as the input to Hierarchical RNN, the human skeleton was divided into five parts according to the physical structure of the human body, and then they were fed into five subnets, respectively. As the number of layers increases, the representation extracted by the subnet merges as higher layer output [106]. Through the distance of the joints and occupancy information of the skeleton, the time information was also extracted using the time pyramid to form the dataset of each action [107,108]. To recognize human actions, the difference in values between consecutive frames was used to calculate the new positions and angles of all joints. The input of the structure tree neural network was the human joints, and the output was the action classification [109]. Zhang et al. [110] extracted the local surface geometric feature (LSGF) of each joint in the point cloud, and introduced the global feature of the vector encoding video sequence. Finally, the SVM classifier was applied to reach the result of the action classification. Khokhova et al. [111] utilized a regular grid to divide the 3D space, and then used descriptors based on space occupancy information to identify the pose of the static frame. An enhanced skeleton visualization method was present [112], in which a CNN was implemented as the main structure to recognize the view-invariant human action.

Human–robot motion retargeting is one of interesting research in human–computer interaction technology. The goal of human–robot motion retargeting is to make the robot follow the movement of the human body. Wang et al. [113,114] established a model as a bridge between the input point cloud of the human body and the robot, so as to achieve the purpose of human–robot motion retargeting. The activity was decomposed into multiple unit sequences, each unit was related to an important factor of behavior [115], and then was inputted into a dynamic Bayesian network to analyze human behavior intentions and realize human–computer interaction.

In addition to some of the above applications, Kim et al. [116] used high-speed RGB and depth sensors to generate movement data of an expert dancer; all skeletons could be reorganized to generate desired dance movements. Given the visual input, let the robot ratiocinate and choose the best container and human pose to perform a transfer task [117]. Soft biometrics can solve the problem of people re-identification. For each measured subject, the 3D skeleton information was applied to adjust the human pose and create the standard pose (SSP) of the skeleton. The SSP was divided into grids to obtain individual characteristics for identification [118]. In archaeology, skeleton joints are helpful to generate a model that can represent any biological shape [119]. The length and position of joints were also beneficial to judge whether the age of the human body is a child or an adult [120]. Desai et al. [121] used the direction of the foot or torso to judge the orientation of the human, and then combined and optimized the skeletons collected by multiple cameras to obtain the final skeleton, even in the case of occlusion. In terms of rehabilitation treatment, a method was advanced to improve the evaluation of upper-limb rehabilitation [122]. The skeleton of the point cloud was taken by Microsoft Kinect, which was registered with the SMPL template to obtain the position and length of the joint.

## 6. Conclusions

In this paper, we review recent researches on the point cloud-based joint extraction of human body. The superiority of point cloud data as well as the applications of joint estimation are all discussed in details. Different works are introduced based on three mainstream methods: (1) template-based methods; (2) feature-based methods; (3) machine learning-based methods. On this basis, we analyze and summarize the current human pose dataset with point cloud. Although a lot of research devotes to the construct the practical pose dataset of human body, there is still a lack of comprehensive ground truth datasets for human varied pose, especially the marking of human joints with different clothes. The relevant applications of point cloud-based joint estimation of human body are discussed in this paper, we found that point cloud-based method plays an important role in some emerging technologies, such as 3D reconstruction, human–computer interaction, action recognition, etc.

From the analysis above, we know that many existing methods have already accurately tracked the human body joints in real time under the indoor environment. However, joint estimation of human body yet still faces many challenges. In our opinion, feature-based method cannot further improve the accuracy of joint detection, if it relies only on the depth features and length constraints of the joints. Therefore, combining with other data from multiple sensors has become a new breakthrough, such as RGB cameras, infrared cameras and IMU sensors. The template-based method and the machine learning-based method are currently unable to recognize the joints of any pose, because neither the template dataset nor the training set can cover all the poses. The 3D template of the human body can be constructed by some software, but there are some limitations on the fixed pose of the template, and the matching process takes a long time. At present, additional information can be leveraged to shorten the search time, it is also possible to build models with better resolution. They will still be interesting research directions in the coming future. In order to improve the detection accuracy, the machine learning-based network should output the local and global features between the points in real time when the input is an orderless point cloud. In addition, there are still some unresolved challenges and gaps between research and practical applications in the entire research field, such as self-occlusion and multi-person detection. However, with the deeper research of machine learning technology, pose estimation of the human body will also be faster and more accurate. Effective networks and sufficient train data are key elements in machine learning-based methods; it is believed that there will be more room for improvement by many scientific researchers investing time in the future. 

## Figures and Tables

**Table 1:** Comparison of different depth cameras.

Depth Cameras	Binocular Stereo Vision	ToF	Structured Light
Advantages	low hardware requirements	long detection distance	convenience for miniaturization
	low cost	large tolerance to ambient range	low resource consumption
	high robustness to light disturbance	high frame rate	high resolution
Disadvantages	large calculation complexity	high equipment requirements	small tolerance to ambient light
	strong object texture dependence	high resource consumption	short detection range
	limited measurement range	low edge accuracy	high noise
Representative	ZED 2K Stereo Camera	Kinect v2	Kinect v1
	BumbleBee	Intel RealSense L515	XTion

**Table 2:** Summary of the referenced works for human pose estimation with depth inputs.

Methods	Dataset	*δ* (/cm)	FPS	GPU
[11]	SMMC-10	9	25	N
[18]	EVAL	10	30	Y
[24]	SMMC-10	10	20	N
[89]	SMMC-10	10	125	N
[44]	SMMC-10	6	25	N
[45]	Self-built dataset	-	-	-
[50]	Self-built dataset	6	-	-
[56]	SMMC-10	15	25	N
[57]	Self-built dataset	6	-	N
[68]	EVAL	10	-	Y
[82]	CMU Mocap	-	-	Y
[87]	Self-built dataset	10	35	N

“-” represents that the value is not given; *δ* represents threshold value; “Y” means running on a desktop with GPU; and “N” means running on a desktop without GPU.

**Table 3:** Tracking accuracy of the human body joints in the referenced works.

	Head	Neck	Shoulders	Elbows	Wrists	Hand	Ankles	Knees	Foot
[11]	0.97	-	-	-	-	0.85	-	-	-
[18]	0.91	-	0.94	0.905	0.818	-	0.93	0.955	-
[24]	0.99	-	0.965	0.965	0.965	-	0.97	0.958	-
[89]	0.975	0.965	0.985	0.96	0.95	-	0.965	0.963	-
[44]	0.96	-	0.995	0.774	-	0.933	-	-	0.99
[45]	0.92	-	-	-	-	0.852	-	-	0.869
[50]	-	0.813	0.882	0.867	-	-	-	0.85	
[56]	0.97	-	0.935	0.86	-	0.885	-	0.935	0.935
[57]	0.979	0.701	0.947	0.926	-	0.754	-	0.463	0.855
[68]	0.917	0.955	0.919	0.763	-	0.839	-	0.852	0.927
[82]	0.97	-	0.955	0.915	0.867	-	0.930	0.925	-
[87]	0.99	-	0.975	0.96	0.95	-	0.965	0.98	-

“-” represents that the value is not given.

**Table 4:** Previously developed depth datasets for human bodies.

Dataset Name	Acquisition Device	Year	Joints	Subjects	Classes	FPS
SMMC-10 [90]	MoCap+ToF	2010	15	S	-	25
MSR-Action3D [91]	Kinect v1	2010	20	S	20	15
NTU RGB+D [92]	Kinect v2	2016	25	M	60	30
Walking gait [93]	Kinect v2	2018	25	S	9	30
MHAD [94]	MoCap+Kinect v1	2013	21	S	11	30
G3D [95]	Kinect v1	2012	20	S	20	30
G3Di [96]	Kinect v1	2014	20	M	18	30
SBU-Kinect-Interaction [97]	Kinect v1	2012	15	M	8	15
CDC4CNV [98]	Kinect v1	2011	9	S	-	-
EVAL [89]	Vicon motion	2012	12	S	24	30
CMU MoCap [99]	MoCap	-	41	M	23	-

S denotes single person, M denotes multi-person interaction. “-” represents that the value is not given.

## Data Availability

No new data were created or analyzed in this study. Data sharing is not applicable to this article.
